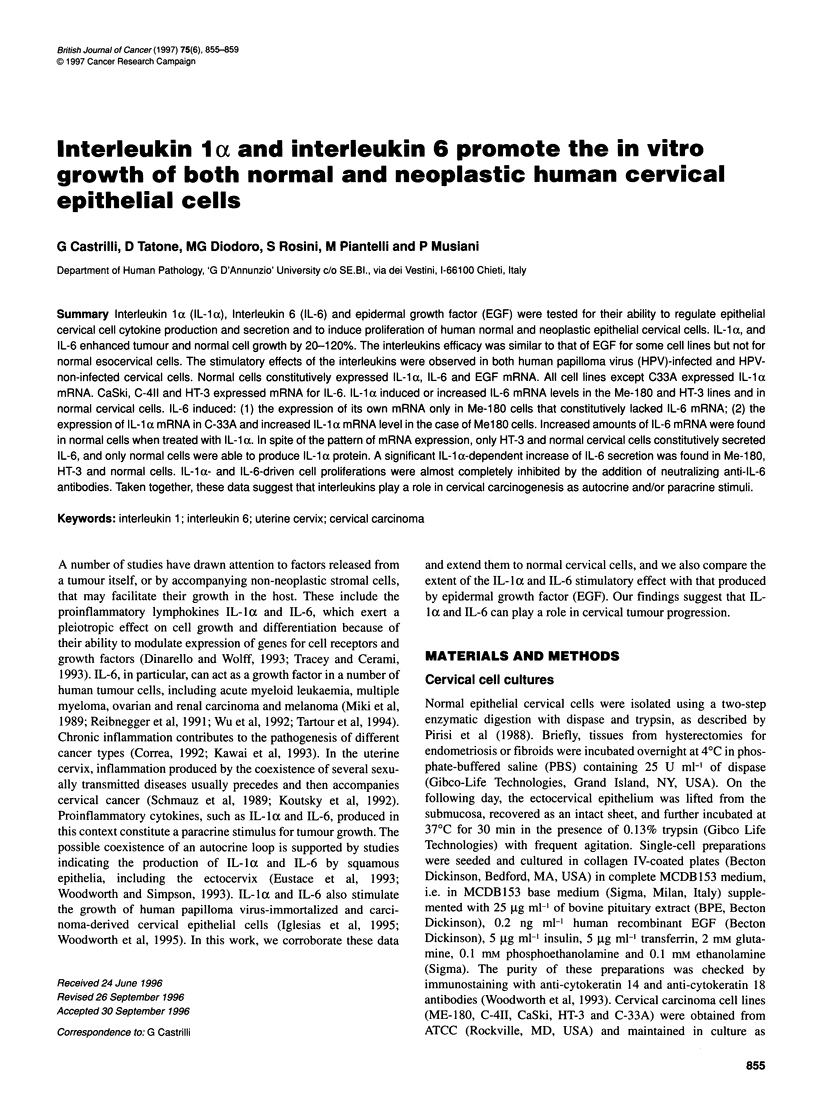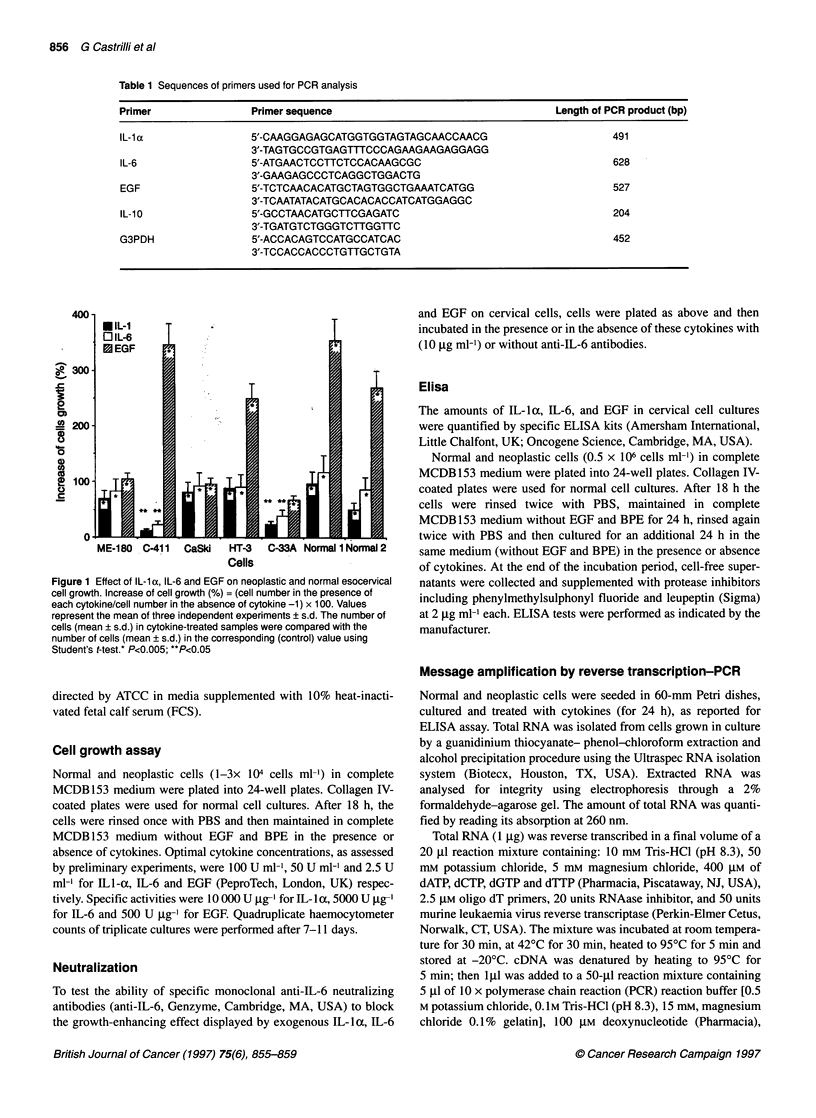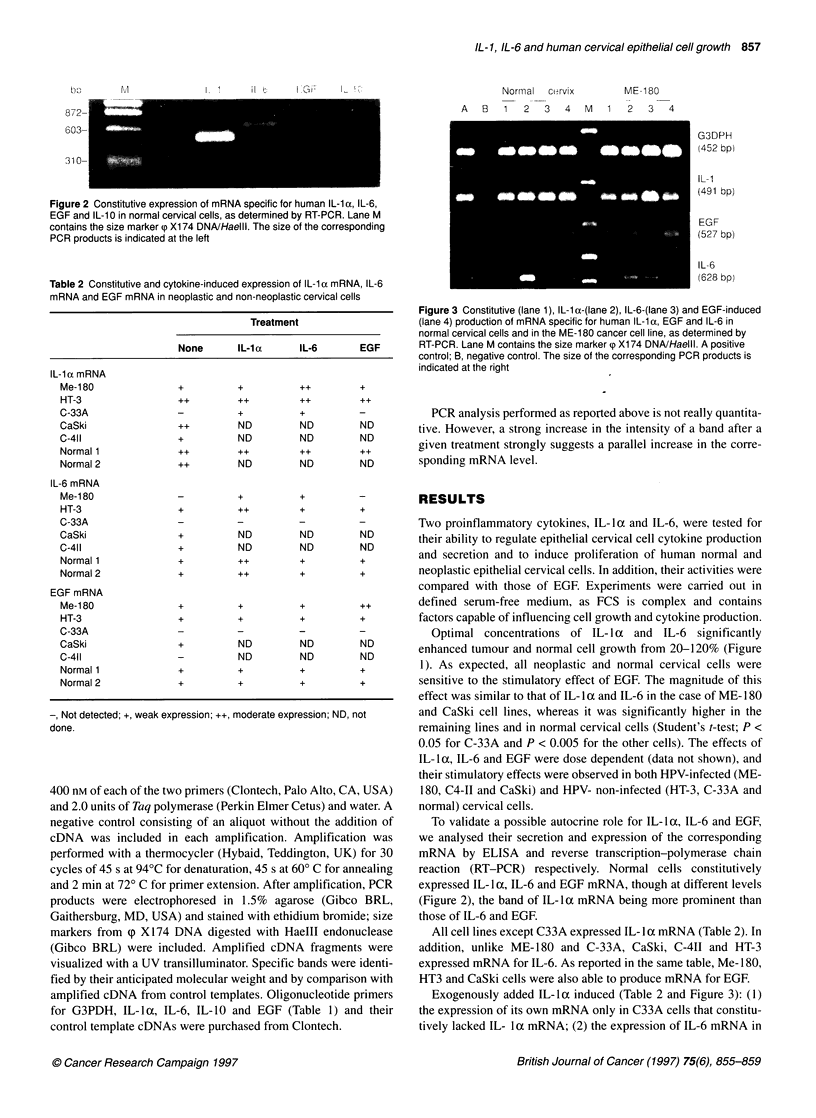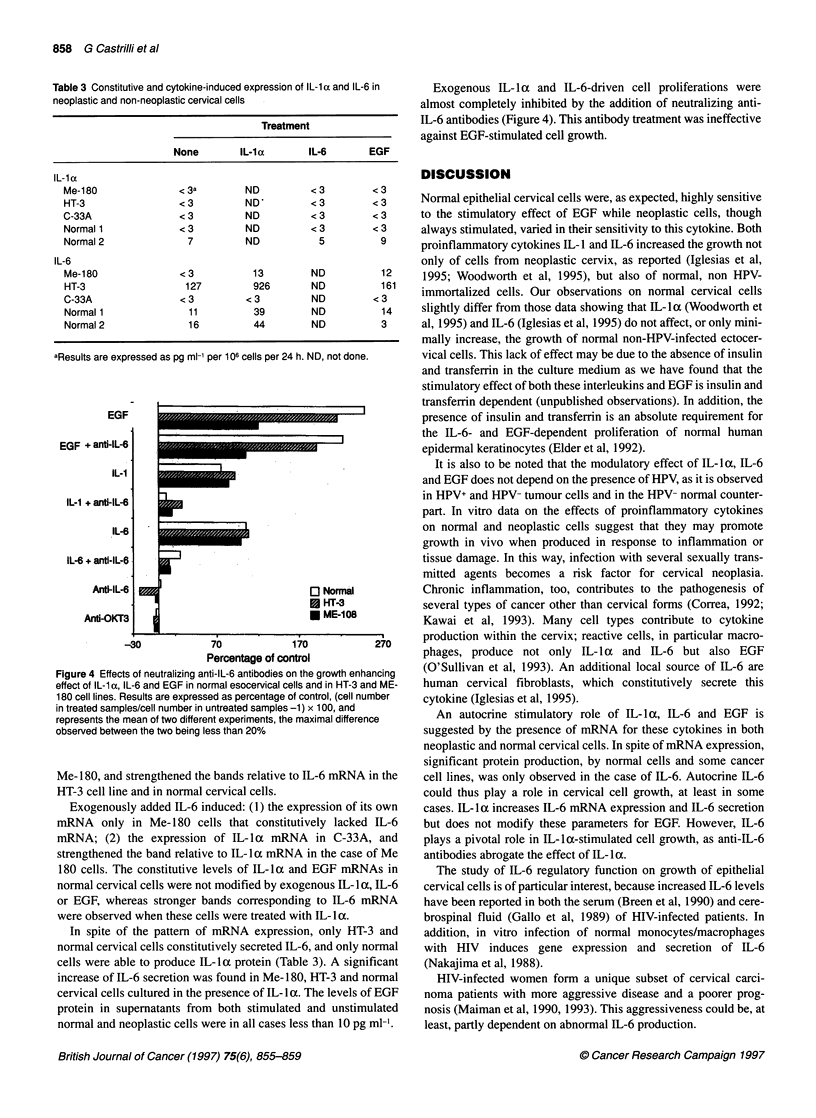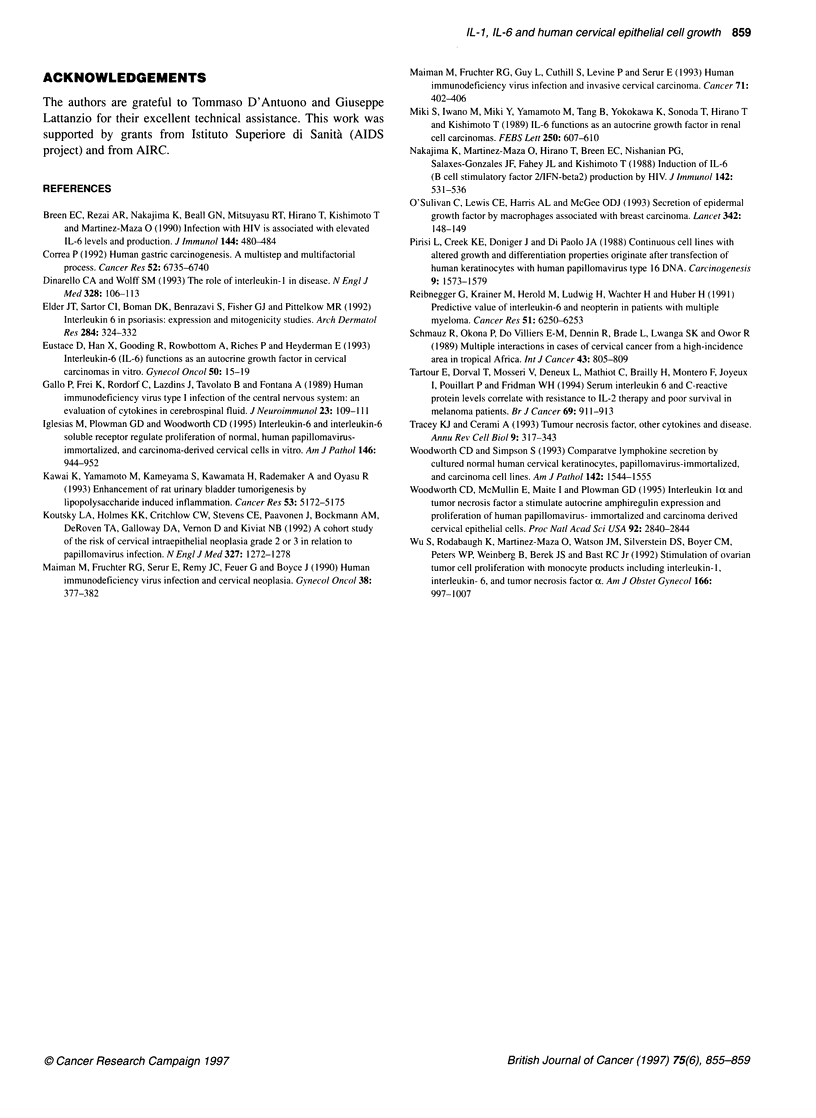# Interleukin 1alpha and interleukin 6 promote the in vitro growth of both normal and neoplastic human cervical epithelial cells.

**DOI:** 10.1038/bjc.1997.152

**Published:** 1997

**Authors:** G. Castrilli, D. Tatone, M. G. Diodoro, S. Rosini, M. Piantelli, P. Musiani

**Affiliations:** Department of Human Pathology, G D'Annuzio University, Chieti, Italy.

## Abstract

**Images:**


British Journal of Cancer (1997) 75(6), 855–859
© 1997 Cancer Research Campaign

### Interleukin 1α and interleukin 6 promote the in vitro growth of both normal and neoplastic human cervical epithelial cells

**G Castrilli, D Tatone, MG Diodoro, S Rosini, M Piantelli and P Musiani**

Department of Human Pathology, 'G D'Annunzio' University c/o SE.BI., via dei Vestini, I-66100 Chieti, Italy

**Summary** Interleukin 1α (IL-1α), Interleukin 6 (IL-6) and epidermal growth factor (EGF) were tested for their ability to regulate epithelial cervical cell cytokine production and secretion and to induce proliferation of human normal and neoplastic epithelial cervical cells. IL-1α, and IL-6 enhanced tumour and normal cell growth by 20–120%. The interleukins efficacy was similar to that of EGF for some cell lines but not for normal esocervical cells. The stimulatory effects of the interleukins were observed in both human papilloma virus (HPV)-infected and HPV-non-infected cervical cells. Normal cells constitutively expressed IL-1α, IL-6 and EGF mRNA. All cell lines except C33A expressed IL-1α mRNA. CaSki, C-4II and HT-3 expressed mRNA for IL-6. IL-1α induced or increased IL-6 mRNA levels in the Me-180 and HT-3 lines and in normal cervical cells. IL-6 induced: (1) the expression of its own mRNA only in Me-180 cells that constitutively lacked IL-6 mRNA; (2) the expression of IL-1α mRNA in C-33A and increased IL-1α mRNA level in the case of Me180 cells. Increased amounts of IL-6 mRNA were found in normal cells when treated with IL-1α. In spite of the pattern of mRNA expression, only HT-3 and normal cervical cells constitutively secreted IL-6, and only normal cells were able to produce IL-1α protein. A significant IL-1α-dependent increase of IL-6 secretion was found in Me-180, HT-3 and normal cells. IL-1α- and IL-6-driven cell proliferations were almost completely inhibited by the addition of neutralizing anti-IL-6 antibodies. Taken together, these data suggest that interleukins play a role in cervical carcinogenesis as autocrine and/or paracrine stimuli.

**Keywords:** interleukin 1; interleukin 6; uterine cervix; cervical carcinoma

A number of studies have drawn attention to factors released from a tumour itself, or by accompanying non-neoplastic stromal cells, that may facilitate their growth in the host. These include the proinflammatory lymphokines IL-1α and IL-6, which exert a pleiotropic effect on cell growth and differentiation because of their ability to modulate expression of genes for cell receptors and growth factors (Dinarello and Wolff, 1993; Tracey and Cerami, 1993). IL-6, in particular, can act as a growth factor in a number of human tumour cells, including acute myeloid leukaemia, multiple myeloma, ovarian and renal carcinoma and melanoma (Miki et al, 1989; Reibnegger et al, 1991; Wu et al, 1992; Tartour et al, 1994). Chronic inflammation contributes to the pathogenesis of different cancer types (Correa, 1992; Kawai et al, 1993). In the uterine cervix, inflammation produced by the coexistence of several sexually transmitted diseases usually precedes and then accompanies cervical cancer (Schmauz et al, 1989; Koutsky et al, 1992). Proinflammatory cytokines, such as IL-1α and IL-6, produced in this context constitute a paracrine stimulus for tumour growth. The possible coexistence of an autocrine loop is supported by studies indicating the production of IL-1α and IL-6 by squamous epithelia, including the ectocervix (Eustace et al, 1993; Woodworth and Simpson, 1993). IL-1α and IL-6 also stimulate the growth of human papilloma virus-immortalized and carcinoma-derived cervical epithelial cells (Iglesias et al, 1995; Woodworth et al, 1995). In this work, we corroborate these data and extend them to normal cervical cells, and we also compare the extent of the IL-1α and IL-6 stimulatory effect with that produced by epidermal growth factor (EGF). Our findings suggest that IL-1α and IL-6 can play a role in cervical tumour progression.

*Received 24 June 1996*
*Revised 26 September 1996*
*Accepted 30 September 1996*

*Correspondence to:* G Castrilli

### MATERIALS AND METHODS

### Cervical cell cultures

Normal epithelial cervical cells were isolated using a two-step enzymatic digestion with dispase and trypsin, as described by Pirisi et al (1988). Briefly, tissues from hysterectomies for endometriosis or fibroids were incubated overnight at 4°C in phosphate-buffered saline (PBS) containing 25 U ml$^{-1}$ of dispase (Gibco-Life Technologies, Grand Island, NY, USA). On the following day, the ectocervical epithelium was lifted from the submucosa, recovered as an intact sheet, and further incubated at 37°C for 30 min in the presence of 0.13% trypsin (Gibco Life Technologies) with frequent agitation. Single-cell preparations were seeded and cultured in collagen IV-coated plates (Becton Dickinson, Bedford, MA, USA) in complete MCDB153 medium, i.e. in MCDB153 base medium (Sigma, Milan, Italy) supplemented with 25 μg ml$^{-1}$ of bovine pituitary extract (BPE, Becton Dickinson), 0.2 ng ml$^{-1}$ human recombinant EGF (Becton Dickinson), 5 μg ml$^{-1}$ insulin, 5 μg ml$^{-1}$ transferrin, 2 mM glutamine, 0.1 mM phosphoethanolamine and 0.1 mM ethanolamine (Sigma). The purity of these preparations was checked by immunostaining with anti-cytokeratin 14 and anti-cytokeratin 18 antibodies (Woodworth et al, 1993). Cervical carcinoma cell lines (ME-180, C-4II, CaSki, HT-3 and C-33A) were obtained from ATCC (Rockville, MD, USA) and maintained in culture as

**Table 1** Sequences of primers used for PCR analysis

| Primer | Primer sequence | Length of PCR product (bp) |
|---|---|---|
| IL-1α | 5′-CAAGGAGAGCATGGTGGTAGTAGCAACCAACG<br>3′-TAGTGCCGTGAGTTTCCCAGAAGAAGAGGAGG | 491 |
| IL-6 | 5′-ATGAACTCCTTCTCCACAAGCGC<br>3′-GAAGAGCCCTCAGGCTGGACTG | 628 |
| EGF | 5′-TCTCAACACATGCTAGTGGCTGAAATCATGG<br>3′-TCAATATACATGCACACACCATCATGGAGGC | 527 |
| IL-10 | 5′-GCCTAACATGCTTCGAGATC<br>3′-TGATGTCTGGGTCTTGGTTC | 204 |
| G3PDH | 5′-ACCACAGTCCATGCCATCAC<br>3′-TCCACCACCCTGTTGCTGTA | 452 |

**Figure 1** Effect of IL-1α, IL-6 and EGF on neoplastic and normal esocervical cell growth. Increase of cell growth (%) = (cell number in the presence of each cytokine/cell number in the absence of cytokine −1) × 100. Values represent the mean of three independent experiments ± s.d. The number of cells (mean ± s.d.) in cytokine-treated samples were compared with the number of cells (mean ± s.d.) in the corresponding (control) value using Student's *t*-test.* *P*<0.005; ***P*<0.05

directed by ATCC in media supplemented with 10% heat-inactivated fetal calf serum (FCS).

### Cell growth assay

Normal and neoplastic cells (1–3× $10^4$ cells $ml^{-1}$) in complete MCDB153 medium were plated into 24-well plates. Collagen IV-coated plates were used for normal cell cultures. After 18 h, the cells were rinsed once with PBS and then maintained in complete MCDB153 medium without EGF and BPE in the presence or absence of cytokines. Optimal cytokine concentrations, as assessed by preliminary experiments, were 100 U $ml^{-1}$, 50 U $ml^{-1}$ and 2.5 U $ml^{-1}$ for IL1-α, IL-6 and EGF (PeproTech, London, UK) respectively. Specific activities were 10 000 U $\mu g^{-1}$ for IL-1α, 5000 U $\mu g^{-1}$ for IL-6 and 500 U $\mu g^{-1}$ for EGF. Quadruplicate haemocytometer counts of triplicate cultures were performed after 7–11 days.

### Neutralization

To test the ability of specific monoclonal anti-IL-6 neutralizing antibodies (anti-IL-6, Genzyme, Cambridge, MA, USA) to block the growth-enhancing effect displayed by exogenous IL-1α, IL-6 and EGF on cervical cells, cells were plated as above and then incubated in the presence or in the absence of these cytokines with (10 μg $ml^{-1}$) or without anti-IL-6 antibodies.

### Elisa

The amounts of IL-1α, IL-6, and EGF in cervical cell cultures were quantified by specific ELISA kits (Amersham International, Little Chalfont, UK; Oncogene Science, Cambridge, MA, USA).

Normal and neoplastic cells (0.5 × $10^6$ cells $ml^{-1}$) in complete MCDB153 medium were plated into 24-well plates. Collagen IV-coated plates were used for normal cell cultures. After 18 h the cells were rinsed twice with PBS, maintained in complete MCDB153 medium without EGF and BPE for 24 h, rinsed again twice with PBS and then cultured for an additional 24 h in the same medium (without EGF and BPE) in the presence or absence of cytokines. At the end of the incubation period, cell-free supernatants were collected and supplemented with protease inhibitors including phenylmethylsulphonyl fluoride and leupeptin (Sigma) at 2 μg $ml^{-1}$ each. ELISA tests were performed as indicated by the manufacturer.

### Message amplification by reverse transcription–PCR

Normal and neoplastic cells were seeded in 60-mm Petri dishes, cultured and treated with cytokines (for 24 h), as reported for ELISA assay. Total RNA was isolated from cells grown in culture by a guanidinium thiocyanate– phenol–chloroform extraction and alcohol precipitation procedure using the Ultraspec RNA isolation system (Biotecx, Houston, TX, USA). Extracted RNA was analysed for integrity using electrophoresis through a 2% formaldehyde–agarose gel. The amount of total RNA was quantified by reading its absorption at 260 nm.

Total RNA (1 μg) was reverse transcribed in a final volume of a 20 μl reaction mixture containing: 10 mM Tris-HCl (pH 8.3), 50 mM potassium chloride, 5 mM magnesium chloride, 400 μM of dATP, dCTP, dGTP and dTTP (Pharmacia, Piscataway, NJ, USA), 2.5 μM oligo dT primers, 20 units RNAase inhibitor, and 50 units murine leukaemia virus reverse transcriptase (Perkin-Elmer Cetus, Norwalk, CT, USA). The mixture was incubated at room temperature for 30 min, at 42°C for 30 min, heated to 95°C for 5 min and stored at −20°C. cDNA was denatured by heating to 95°C for 5 min; then 1μl was added to a 50-μl reaction mixture containing 5 μl of 10 × polymerase chain reaction (PCR) reaction buffer [0.5 M potassium chloride, 0.1M Tris-HCl (pH 8.3), 15 mM, magnesium chloride 0.1% gelatin], 100 μM deoxynucleotide (Pharmacia),

 © Cancer Research Campaign 1997

**Figure 2** Constitutive expression of mRNA specific for human IL-1α, IL-6, EGF and IL-10 in normal cervical cells, as determined by RT-PCR. Lane M contains the size marker φ X174 DNA/*Hae*III. The size of the corresponding PCR products is indicated at the left

**Table 2** Constitutive and cytokine-induced expression of IL-1α mRNA, IL-6 mRNA and EGF mRNA in neoplastic and non-neoplastic cervical cells

| | Treatment | | | |
|---|---|---|---|---|
| | **None** | **IL-1α** | **IL-6** | **EGF** |
| IL-1α mRNA | | | | |
| Me-180 | + | + | ++ | + |
| HT-3 | ++ | ++ | ++ | ++ |
| C-33A | – | + | + | – |
| CaSki | ++ | ND | ND | ND |
| C-4II | + | ND | ND | ND |
| Normal 1 | ++ | ++ | ++ | ++ |
| Normal 2 | ++ | ND | ND | ND |
| IL-6 mRNA | | | | |
| Me-180 | – | + | + | – |
| HT-3 | + | ++ | + | + |
| C-33A | – | – | – | – |
| CaSki | + | ND | ND | ND |
| C-4II | + | ND | ND | ND |
| Normal 1 | + | ++ | + | + |
| Normal 2 | + | ++ | + | + |
| EGF mRNA | | | | |
| Me-180 | + | + | + | ++ |
| HT-3 | + | + | + | + |
| C-33A | – | – | – | – |
| CaSki | + | ND | ND | ND |
| C-4II | – | ND | ND | ND |
| Normal 1 | + | + | + | + |
| Normal 2 | + | + | + | + |

–, Not detected; +, weak expression; ++, moderate expression; ND, not done.

400 nM of each of the two primers (Clontech, Palo Alto, CA, USA) and 2.0 units of *Taq* polymerase (Perkin Elmer Cetus) and water. A negative control consisting of an aliquot without the addition of cDNA was included in each amplification. Amplification was performed with a thermocycler (Hybaid, Teddington, UK) for 30 cycles of 45 s at 94°C for denaturation, 45 s at 60° C for annealing and 2 min at 72° C for primer extension. After amplification, PCR products were electrophoresed in 1.5% agarose (Gibco BRL, Gaithersburg, MD, USA) and stained with ethidium bromide; size markers from φ X174 DNA digested with HaeIII endonuclease (Gibco BRL) were included. Amplified cDNA fragments were visualized with a UV transilluminator. Specific bands were identified by their anticipated molecular weight and by comparison with amplified cDNA from control templates. Oligonucleotide primers for G3PDH, IL-1α, IL-6, IL-10 and EGF (Table 1) and their control template cDNAs were purchased from Clontech.

**Figure 3** Constitutive (lane 1), IL-1α-(lane 2), IL-6-(lane 3) and EGF-induced (lane 4) production of mRNA specific for human IL-1α, EGF and IL-6 in normal cervical cells and in the ME-180 cancer cell line, as determined by RT-PCR. Lane M contains the size marker φ X174 DNA/*Hae*III. A positive control; B, negative control. The size of the corresponding PCR products is indicated at the right

PCR analysis performed as reported above is not really quantitative. However, a strong increase in the intensity of a band after a given treatment strongly suggests a parallel increase in the corresponding mRNA level.

### RESULTS

Two proinflammatory cytokines, IL-1α and IL-6, were tested for their ability to regulate epithelial cervical cell cytokine production and secretion and to induce proliferation of human normal and neoplastic epithelial cervical cells. In addition, their activities were compared with those of EGF. Experiments were carried out in defined serum-free medium, as FCS is complex and contains factors capable of influencing cell growth and cytokine production.

Optimal concentrations of IL-1α and IL-6 significantly enhanced tumour and normal cell growth from 20–120% (Figure 1). As expected, all neoplastic and normal cervical cells were sensitive to the stimulatory effect of EGF. The magnitude of this effect was similar to that of IL-1α and IL-6 in the case of ME-180 and CaSki cell lines, whereas it was significantly higher in the remaining lines and in normal cervical cells (Student's *t*-test; $P < 0.05$ for C-33A and $P < 0.005$ for the other cells). The effects of IL-1α, IL-6 and EGF were dose dependent (data not shown), and their stimulatory effects were observed in both HPV-infected (ME-180, C4-II and CaSki) and HPV- non-infected (HT-3, C-33A and normal) cervical cells.

To validate a possible autocrine role for IL-1α, IL-6 and EGF, we analysed their secretion and expression of the corresponding mRNA by ELISA and reverse transcription–polymerase chain reaction (RT–PCR) respectively. Normal cells constitutively expressed IL-1α, IL-6 and EGF mRNA, though at different levels (Figure 2), the band of IL-1α mRNA being more prominent than those of IL-6 and EGF.

All cell lines except C33A expressed IL-1α mRNA (Table 2). In addition, unlike ME-180 and C-33A, CaSki, C-4II and HT-3 expressed mRNA for IL-6. As reported in the same table, Me-180, HT3 and CaSki cells were also able to produce mRNA for EGF.

Exogenously added IL-1α induced (Table 2 and Figure 3): (1) the expression of its own mRNA only in C33A cells that constitutively lacked IL- 1α mRNA; (2) the expression of IL-6 mRNA in

© Cancer Research Campaign 1997

**Table 3** Constitutive and cytokine-induced expression of IL-1α and IL-6 in neoplastic and non-neoplastic cervical cells

| | Treatment | | | |
|---|---|---|---|---|
| | None | IL-1α | IL-6 | EGF |
| IL-1α | | | | |
| Me-180 | < 3[a] | ND | < 3 | < 3 |
| HT-3 | < 3 | ND | < 3 | < 3 |
| C-33A | < 3 | ND | < 3 | < 3 |
| Normal 1 | < 3 | ND | < 3 | < 3 |
| Normal 2 | 7 | ND | 5 | 9 |
| IL-6 | | | | |
| Me-180 | < 3 | 13 | ND | 12 |
| HT-3 | 127 | 926 | ND | 161 |
| C-33A | < 3 | < 3 | ND | < 3 |
| Normal 1 | 11 | 39 | ND | 14 |
| Normal 2 | 16 | 44 | ND | 3 |

[a]Results are expressed as pg $ml^{-1}$ per $10^6$ cells per 24 h. ND, not done.

**Figure 4** Effects of neutralizing anti-IL-6 antibodies on the growth enhancing effect of IL-1α, IL-6 and EGF in normal esocervical cells and in HT-3 and ME-180 cell lines. Results are expressed as percentage of control, (cell number in treated samples/cell number in untreated samples −1) × 100, and represents the mean of two different experiments, the maximal difference observed between the two being less than 20%

Me-180, and strengthened the bands relative to IL-6 mRNA in the HT-3 cell line and in normal cervical cells.

Exogenously added IL-6 induced: (1) the expression of its own mRNA only in Me-180 cells that constitutively lacked IL-6 mRNA; (2) the expression of IL-1α mRNA in C-33A, and strengthened the band relative to IL-1α mRNA in the case of Me 180 cells. The constitutive levels of IL-1α and EGF mRNAs in normal cervical cells were not modified by exogenous IL-1α, IL-6 or EGF, whereas stronger bands corresponding to IL-6 mRNA were observed when these cells were treated with IL-1α.

In spite of the pattern of mRNA expression, only HT-3 and normal cervical cells constitutively secreted IL-6, and only normal cells were able to produce IL-1α protein (Table 3). A significant increase of IL-6 secretion was found in Me-180, HT-3 and normal cervical cells cultured in the presence of IL-1α. The levels of EGF protein in supernatants from both stimulated and unstimulated normal and neoplastic cells were in all cases less than 10 pg $ml^{-1}$.

Exogenous IL-1α and IL-6-driven cell proliferations were almost completely inhibited by the addition of neutralizing anti-IL-6 antibodies (Figure 4). This antibody treatment was ineffective against EGF-stimulated cell growth.

### DISCUSSION

Normal epithelial cervical cells were, as expected, highly sensitive to the stimulatory effect of EGF while neoplastic cells, though always stimulated, varied in their sensitivity to this cytokine. Both proinflammatory cytokines IL-1 and IL-6 increased the growth not only of cells from neoplastic cervix, as reported (Iglesias et al, 1995; Woodworth et al, 1995), but also of normal, non HPV-immortalized cells. Our observations on normal cervical cells slightly differ from those data showing that IL-1α (Woodworth et al, 1995) and IL-6 (Iglesias et al, 1995) do not affect, or only minimally increase, the growth of normal non-HPV-infected ectocervical cells. This lack of effect may be due to the absence of insulin and transferrin in the culture medium as we have found that the stimulatory effect of both these interleukins and EGF is insulin and transferrin dependent (unpublished observations). In addition, the presence of insulin and transferrin is an absolute requirement for the IL-6- and EGF-dependent proliferation of normal human epidermal keratinocytes (Elder et al, 1992).

It is also to be noted that the modulatory effect of IL-1α, IL-6 and EGF does not depend on the presence of HPV, as it is observed in $HPV^+$ and $HPV^-$ tumour cells and in the $HPV^-$ normal counterpart. In vitro data on the effects of proinflammatory cytokines on normal and neoplastic cells suggest that they may promote growth in vivo when produced in response to inflammation or tissue damage. In this way, infection with several sexually transmitted agents becomes a risk factor for cervical neoplasia. Chronic inflammation, too, contributes to the pathogenesis of several types of cancer other than cervical forms (Correa, 1992; Kawai et al, 1993). Many cell types contribute to cytokine production within the cervix; reactive cells, in particular macrophages, produce not only IL-1α and IL-6 but also EGF (O'Sullivan et al, 1993). An additional local source of IL-6 are human cervical fibroblasts, which constitutively secrete this cytokine (Iglesias et al, 1995).

An autocrine stimulatory role of IL-1α, IL-6 and EGF is suggested by the presence of mRNA for these cytokines in both neoplastic and normal cervical cells. In spite of mRNA expression, significant protein production, by normal cells and some cancer cell lines, was only observed in the case of IL-6. Autocrine IL-6 could thus play a role in cervical cell growth, at least in some cases. IL-1α increases IL-6 mRNA expression and IL-6 secretion but does not modify these parameters for EGF. However, IL-6 plays a pivotal role in IL-1α-stimulated cell growth, as anti-IL-6 antibodies abrogate the effect of IL-1α.

The study of IL-6 regulatory function on growth of epithelial cervical cells is of particular interest, because increased IL-6 levels have been reported in both the serum (Breen et al, 1990) and cerebrospinal fluid (Gallo et al, 1989) of HIV-infected patients. In addition, in vitro infection of normal monocytes/macrophages with HIV induces gene expression and secretion of IL-6 (Nakajima et al, 1988).

HIV-infected women form a unique subset of cervical carcinoma patients with more aggressive disease and a poorer prognosis (Maiman et al, 1990, 1993). This aggressiveness could be, at least, partly dependent on abnormal IL-6 production.

© Cancer Research Campaign 1997

### ACKNOWLEDGEMENTS

The authors are grateful to Tommaso D'Antuono and Giuseppe Lattanzio for their excellent technical assistance. This work was supported by grants from Istituto Superiore di Sanità (AIDS project) and from AIRC.

### REFERENCES

Breen EC, Rezai AR, Nakajima K, Beall GN, Mitsuyasu RT, Hirano T, Kishimoto T and Martinez-Maza O (1990) Infection with HIV is associated with elevated IL-6 levels and production. *J Immunol* **144:** 480–484

Correa P (1992) Human gastric carcinogenesis. A multistep and multifactorial process. *Cancer Res* **52:** 6735–6740

Dinarello CA and Wolff SM (1993) The role of interleukin-1 in disease. *N Engl J Med* **328:** 106–113

Elder JT, Sartor CI, Boman DK, Benrazavi S, Fisher GJ and Pittelkow MR (1992) Interleukin 6 in psoriasis: expression and mitogenicity studies. *Arch Dermatol Res* **284:** 324–332

Eustace D, Han X, Gooding R, Rowbottom A, Riches P and Heyderman E (1993) Interleukin-6 (IL-6) functions as an autocrine growth factor in cervical carcinomas in vitro. *Gynecol Oncol* **50:** 15–19

Gallo P, Frei K, Rordorf C, Lazdins J, Tavolato B and Fontana A (1989) Human immunodeficiency virus type I infection of the central nervous system: an evaluation of cytokines in cerebrospinal fluid. *J Neuroimmunol* **23:** 109–111

Iglesias M, Plowman GD and Woodworth CD (1995) Interleukin-6 and interleukin-6 soluble receptor regulate proliferation of normal, human papillomavirus-immortalized, and carcinoma-derived cervical cells in vitro. *Am J Pathol* **146:** 944–952

Kawai K, Yamamoto M, Kameyama S, Kawamata H, Rademaker A and Oyasu R (1993) Enhancement of rat urinary bladder tumorigenesis by lipopolysaccharide induced inflammation. *Cancer Res* **53:** 5172–5175

Koutsky LA, Holmes KK, Critchlow CW, Stevens CE, Paavonen J, Bockmann AM, DeRoven TA, Galloway DA, Vernon D and Kiviat NB (1992) A cohort study of the risk of cervical intraepithelial neoplasia grade 2 or 3 in relation to papillomavirus infection. *N Engl J Med* **327:** 1272–1278

Maiman M, Fruchter RG, Serur E, Remy JC, Feuer G and Boyce J (1990) Human immunodeficiency virus infection and cervical neoplasia. *Gynecol Oncol* **38:** 377–382

Maiman M, Fruchter RG, Guy L, Cuthill S, Levine P and Serur E (1993) Human immunodeficiency virus infection and invasive cervical carcinoma. *Cancer* **71:** 402–406

Miki S, Iwano M, Miki Y, Yamamoto M, Tang B, Yokokawa K, Sonoda T, Hirano T and Kishimoto T (1989) IL-6 functions as an autocrine growth factor in renal cell carcinomas. *FEBS Lett* **250:** 607–610

Nakajima K, Martinez-Maza O, Hirano T, Breen EC, Nishanian PG, Salaxes-Gonzales JF, Fahey JL and Kishimoto T (1988) Induction of IL-6 (B cell stimulatory factor 2/IFN-beta2) production by HIV. *J Immunol* **142:** 531–536

O'Sulivan C, Lewis CE, Harris AL and McGee ODJ (1993) Secretion of epidermal growth factor by macrophages associated with breast carcinoma. *Lancet* **342:** 148–149

Pirisi L, Creek KE, Doniger J and Di Paolo JA (1988) Continuous cell lines with altered growth and differentiation properties originate after transfection of human keratinocytes with human papillomavirus type 16 DNA. *Carcinogenesis* **9:** 1573–1579

Reibnegger G, Krainer M, Herold M, Ludwig H, Wachter H and Huber H (1991) Predictive value of interleukin-6 and neopterin in patients with multiple myeloma. *Cancer Res* **51:** 6250–6253

Schmauz R, Okona P, Do Villiers E-M, Dennin R, Brade L, Lwanga SK and Owor R (1989) Multiple interactions in cases of cervical cancer from a high-incidence area in tropical Africa. *Int J Cancer* **43:** 805–809

Tartour E, Dorval T, Mosseri V, Deneux L, Mathiot C, Brailly H, Montero F, Joyeux I, Pouillart P and Fridman WH (1994) Serum interleukin 6 and C-reactive protein levels correlate with resistance to IL-2 therapy and poor survival in melanoma patients. *Br J Cancer* **69:** 911–913

Tracey KJ and Cerami A (1993) Tumour necrosis factor, other cytokines and disease. *Annu Rev Cell Biol* **9:** 317–343

Woodworth CD and Simpson S (1993) Comparatve lymphokine secretion by cultured normal human cervical keratinocytes, papillomavirus-immortalized, and carcinoma cell lines. *Am J Pathol* **142:** 1544–1555

Woodworth CD, McMullin E, Maite I and Plowman GD (1995) Interleukin 1α and tumor necrosis factor a stimulate autocrine amphiregulin expression and proliferation of human papillomavirus- immortalized and carcinoma derived cervical epithelial cells. *Proc Natl Acad Sci USA* **92:** 2840–2844

Wu S, Rodabaugh K, Martinez-Maza O, Watson JM, Silverstein DS, Boyer CM, Peters WP, Weinberg B, Berek JS and Bast RC Jr (1992) Stimulation of ovarian tumor cell proliferation with monocyte products including interleukin-1, interleukin- 6, and tumor necrosis factor α. *Am J Obstet Gynecol* **166:** 997–1007

© Cancer Research Campaign 1997